# Redefining the norms: an interdisciplinary perspective on language testing in multilinguals with acquired and progressive neurogenic disorders

**DOI:** 10.1002/dad2.70366

**Published:** 2026-06-08

**Authors:** Alessa Hausmann, Adolfo M. García, Ana Sofia Costa, Arpita Bose, Boon Lead Tee, Claudia Rivera‐Fernandez, Eduardo Europa, Gitit Kavé, Jee Eun Sung, Maira Okada de Oliveira, Mandy Wigdorowitz, Maria Garraffa, Maria Teresa Carthery‐Goulart, Michael J. Kleiman, Naama Friedmann, Samir Diouny, Sanne Franzen, Sofia Toniolo, Stephanie Grasso, Thomas H. Bak, Valentina Borghesani

**Affiliations:** ^1^ Department of Psychology University of Geneva Geneva Switzerland; ^2^ Centro de Neurociencias Cognitivas Universidad de San Andrés Buenos Aires Argentina; ^3^ Global Brain Health Institute (GBHI) University of California San Francisco USA; ^4^ Departamento de Lingüística y Literatura, Facultad de Humanidades Universidad de Santiago de Chile Santiago Chile; ^5^ Department of Neurology University Hospital RWTH Aachen University Aachen Germany; ^6^ School of Psychology and Clinical Language Sciences University of Reading Reading UK; ^7^ UCSF Edward and Pearl Fein Memory and Aging Center University of California at San Francisco San Francisco USA; ^8^ Facultad de Psicología Universidad Complutense de Madrid Madrid Spain; ^9^ Universidad Tecnológica del Perú Arequipa Perú; ^10^ Department of Communicative Disorders and Sciences San José State University San José California USA; ^11^ Department of Education and Psychology The Open University of Israel Ra'anana Israel; ^12^ Department of Communication Disorders Ewha Womans University Seoul South Korea; ^13^ Cognitive Neurology and Behavioral Unit (GNCC) University of São Paulo São Paulo Brazil; ^14^ Modern Languages and Classics University of Alabama Tuscaloosa USA; ^15^ Theoretical and Applied Linguistics University of Cambridge Cambridge UK; ^16^ Department of Psychology University of Johannesburg Johannesburg South Africa; ^17^ Faculty of Medicine and Health Sciences East Anglia University Norwich UK; ^18^ Human Communication, Learning and Development Unit, Faculty of Education The University of Hong Kong Hong Kong SAR; ^19^ Center of Mathematics, Computing, and Cognition Federal University of ABC Santo André Brazil; ^20^ Comprehensive Center for Brain Health University of Miami Miller School of Medicine Boca Raton USA; ^21^ Language and Brain Lab, Sagol School of Neuroscience and School of Education Tel Aviv University Tel Aviv Israel; ^22^ Clinical Neuroscience & Mental Health Lab Hassan II University Casablanca Morocco; ^23^ Department of Neurology & Alzheimer Center Erasmus MC University Medical Center Rotterdam the Netherlands; ^24^ Oxford Health Biomedical Research Centre Oxford UK; ^25^ University of Texas at Austin Austin USA; ^26^ School of Philosophy, Psychology & Language Sciences (PPLS) University of Edinburgh Edinburgh Scotland

**Keywords:** acquired aphasia, brain health equity, language assessment, multilingualism, personalized medicine, progressive neurogenic disorders

## Abstract

Accurate language assessment is essential in clinical and research contexts. However, most assessment tools and questionnaires have been developed within monolingual frameworks, overlooking the particularities of the vast multilingual population and, thus, often leading to misdiagnosis and inequitable care. Here, we examine the limitations of monolingual‐centric approaches to language testing in multilinguals with acquired and progressive neurogenic disorders and explore the implications of multilingualism across linguistic, cognitive, neural, clinical, and sociocultural domains. We also address the need for technological innovation and theoretical refinement to better accommodate linguistic diversity in healthcare. Ultimately, we advocate for a nuanced, equitable, and culturally sensitive model of language assessment that reflects the realities of multilingual populations by accounting for their diverse linguistic repertoires, cognitive trajectories, and neural profiles. This model integrates valid, adapted tools, inclusive normative data, and context‐appropriate testing practices, with particular attention to under‐represented and typologically diverse languages.

## Highlights

- Language tests must reflect multilingual brains, not just monolingual norms.
- Ignoring linguistic, cognitive, or neural factors of multilingualism risks misdiagnosis.
- Tools must fit diverse, global patient realities beyond WEIRD samples.
- A paradigm shift is needed for fair, equitable multilingual language assessment.

## 1 INTRODUCTION: THE CONCEPTUAL AMBIGUITIES OF NAVIGATING MULTILINGUAL MINDS

Accurate language assessment is a cornerstone of clinical and research practices, guiding diagnosis, treatment, and patient‐support decisions. This applies to all neurological conditions, including stroke, traumatic brain injury, neurodegenerative dementias, brain tumors, and neurodevelopmental disorders. The task becomes increasingly complex in multilingual populations, which represent more than half of the global population.^1^ Most societies are linguistically diverse, with more than one language spoken within their territory.^2^ Yet, while multilinguals use several languages in their daily lives, assessment practices largely rely on monolingual tools, creating a mismatch that poses critical challenges in both clinical and research contexts.

Most available language assessments were developed for monolinguals, focusing on English and a few other Germanic and Romance languages.^3^ Moreover, these assessments usually presuppose that all speakers share a single, uniform language profile, which results in biased comparisons when applied to multilingual individuals. This approach overlooks the complexity of multilingualism, partly driven by variability in age of second language (L2) acquisition, proficiency, daily usage, exposure, and switching practices, among other characteristics.^4^ These variables impinge on processes that pervade multilingual cognition, including language mixing, switching, cross‐linguistic priming, interference, and (unconscious) translation. As a result, multilinguals often perform differently on language and cognitive tasks, not because of organic or neurological deficits, but because assessments fail to account for their full language experience and communicative contexts.

This makes multilingual individuals particularly vulnerable to misdiagnosis and inappropriate interventions. For instance, linguistic inadequacies (e.g., driven by cross‐linguistic transfer) may be mistaken for pathological behavior, while intact performance on certain cognitive tasks, such as those assessing executive functions or language, may mask underlying impairments, resulting in inappropriate or delayed diagnosis and, therefore, intervention.^5^ For example, multilingual individuals often generate fewer words in verbal fluency tasks than monolingual norms would predict. This reduced output is frequently misinterpreted as reflecting limited proficiency in the tested language rather than an underlying language or cognitive impairment. As a result, clinicians may conclude that it is unclear whether a true deficit is present, leading to diagnostic uncertainty. Consequently, individuals may not qualify for early interventions such as speech–language therapy or cognitive rehabilitation, and access to diagnostic imaging or pharmacological treatment may be delayed. These risks are compounded by the widespread practice of conducting assessments without proper linguistic support, and in some cases, patients are not even referred for assessment in the first place due to language barriers. Assessments are frequently made in a single language (often the dominant language of the context) and/or without a skilled interpreter if required. This practice has remained largely unchanged across Europe, with 90% of clinicians still relying on family members to serve as interpreters or language brokers.^6^

Compounding these challenges is the lack of a unified definition of multilingualism. For clarification purposes, we will use the term multilingualism rather than bilingualism throughout the paper to refer broadly to individuals who speak more than one language, although research suggests that speaking more than two languages offers additional protective effects against cognitive aging (Pot et al., 2018)^7^, beyond the effect of other variables such as education. While some definitions require native‐like proficiency in two or more languages, others invoke a continuum from passive knowledge to full mastery, amidst massive sociolinguistic, cognitive, and experiential diversity. Considering these methodological, translational, and conceptual challenges, a multilingual‐sensitive framework for language testing necessitates robust collaboration across diverse disciplines, including linguistics, (neuro)psychology, speech‐language pathology, neuroscience, clinical practice, data science, and psychometrics. As multilingualism continues to shape modern societies, education, and cognition, its study remains central to understanding human communication in an increasingly interconnected world.

Given our aim to critically examine why monolingual‐based language testing frameworks fail to capture typical and pathological language performance in multilingual individuals with acquired and progressive forms of aphasia, we conducted a narrative review. This approach was selected to allow a flexible and integrative synthesis of evidence across multiple domains, including linguistics, neuroscience, neurology, neuropsychology, speech and language therapy, and data science. A narrative framework is particularly suited to this objective, as it enables the integration of conceptual, methodological, and clinical perspectives that cannot be readily captured through narrowly predefined inclusion criteria. Relevant literature was identified through targeted searches in PubMed, Embase, and PsycInfo, complemented by reference chaining of key empirical papers, reviews, and international guidelines. The multidisciplinary and multilingual composition of the author team, all members of the International Network for Cross‐Linguistic Research on Brain Health (Include) Network, further informed the selection, interpretation, and contextualization of sources. Collectively, these perspectives guided the formulation of the following research question: *How and why do monolingual‐based language testing norms fail to capture typical and pathological language performance in multilingual individuals with acquired and progressive aphasia, and what interdisciplinary frameworks can guide the development of more valid and equitable assessment practices?*

**FIGURE 1:**
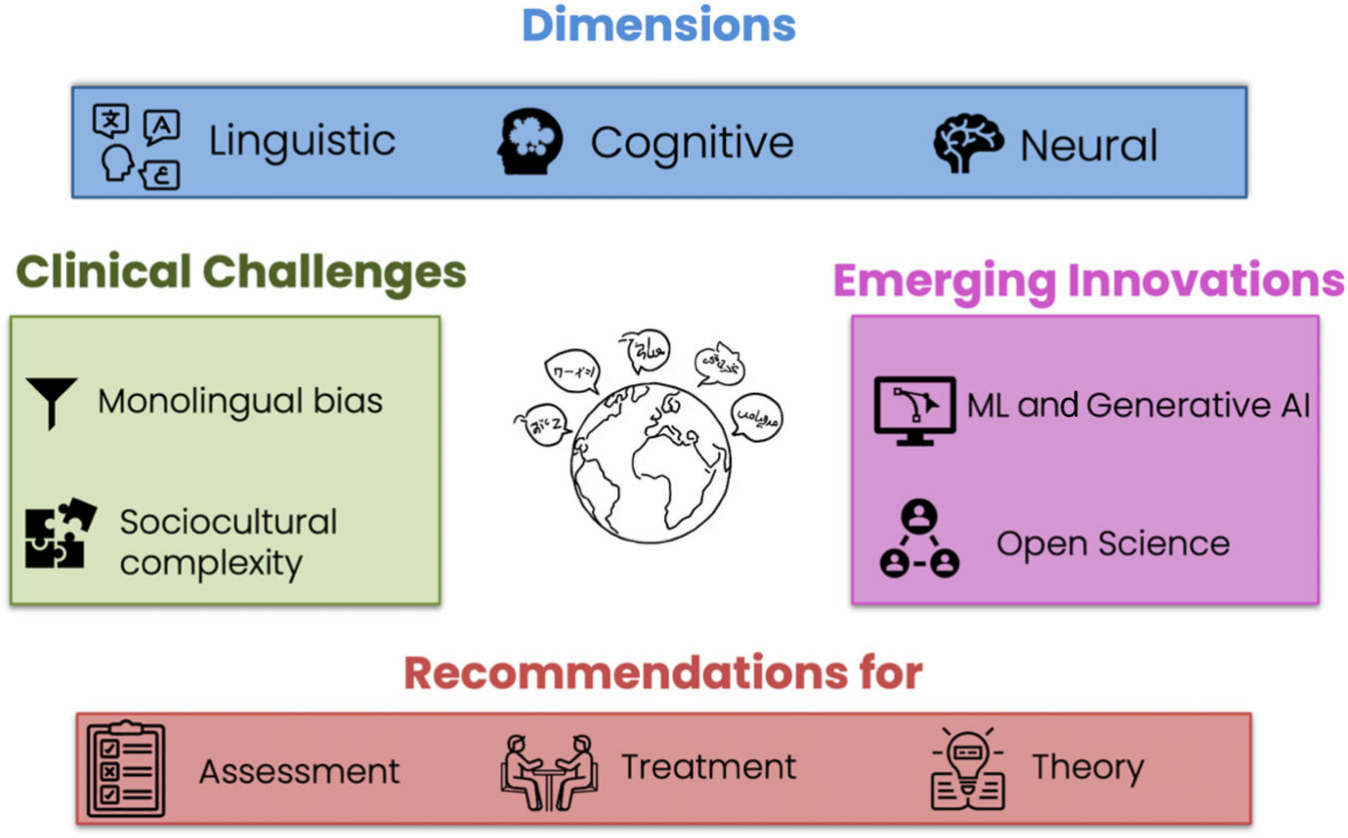
Roadmap of the paper. This figure outlines the organization of the manuscript. It illustrates the logical progression from the conceptual background and rationale, through clinical challenges and emerging innovations, to recommendations for assessment, treatment, and theory.

In line with this research question, this paper surveys core topics to inform the development of a multilingual‐sensitive framework for language assessment in multilingual individuals with acquired and progressive neurogenic disorders (Figure 1). We begin by situating the topic within existing research on the linguistic (Section 2), cognitive (Section 3), and neural (Section 4) dimensions of multilingualism. We then examine the current clinical and research landscapes and their consequences for multilingual assessment (Section 5), along with key intersectional factors that further shape evaluation and care (Sections 6 and 7). Next, we explore the potential of two emerging innovations: machine learning and generative AI (Section 8) and the growing adoption of open science practices (Section 9). We conclude by proposing future directions for both clinical implementation (Section 10) and theoretical advancement (Section 11), as well as recommendations for both clinicians and researchers (Section 12). Overall, we seek to provide a comprehensive roadmap for more inclusive, theory‐founded testing of multilingual individuals.

## 2 LINGUISTIC COMPLEXITY IN MULTILINGUAL ASSESSMENT: STRUCTURE, MODALITY, AND SOCIOLINGUISTIC VARIATION

Assessing multilingual individuals with neurological communication disorders requires a nuanced understanding of how they use and navigate their different languages. This section focuses on key linguistic components and background factors relevant to multilingual assessment. Specifically, we address (1) linguistic typology (i.e., the classification of languages based on their structural features) as a measure of synchronic distance between languages, (2) individuals’ language history and proficiency, and (3) language modality and suprasegmental linguistic features, such as dialects.

A critical aspect of multilingual assessment is recognizing language‐universal and language‐specific features shaped by linguistic typology and reflected across phonology, syntax, semantics, pragmatics, and prosody. These typological factors influence language use and the manifestation of impairments, underscoring the need for tailored assessment approaches. For instance, Spanish–English bilinguals manage two typologically similar languages, both with subject‐verb‐object (SVO) word order and many cognates. In contrast, Korean–English bilinguals navigate distant systems: Korean follows subject‐object‐verb (SOV) order, differs in phonology and orthography, and shares few words with English. Such structural divergences directly affect language use and clinical assessment. The degree of linguistic typological distance between an individual's languages can shape both language symptoms and performance on diagnostic tools.^8^ Understanding these dynamics is particularly critical when language difficulties are not merely developmental or situational but indicate either an acute acquired aphasia or the onset of a progressive neurodegenerative condition.

A key clinical application of these linguistic variables lies in the diagnosis of both acute acquired aphasia and progressive neurodegenerative language disorders, such as primary progressive aphasia (PPA), which often exhibit language‐specific features during assessment.^9^ Linguistic structure appears to shape how PPA variants manifest across languages. In Italian, expressive agrammatism is more common, likely reflecting its rich morphology, whereas English speakers more often show apraxia of speech, possibly due to the language's complex articulatory demands.^10^ Similar language‐specific effects have been observed beyond Indo‐European languages: Speakers with non‐fluent post‐stroke aphasia in West Greenlandic, a polysynthetic language, tend to preserve grammatical accuracy, likely supported by features such as obligatory inflections and fixed sentence constructions.^11^ Likewise, in Bengali speakers with Alzheimer's disease, pronoun‐drop properties and rich inflectional morphology seem to modulate symptom expressions.^12^, ^13^ Together, these findings underscore the value of cross‐linguistic comparisons for identifying robust and clinically meaningful language markers.

Beyond spoken modality, properties of written language also influence symptom expression. Orthographic systems, including spelling‐to‐sound transparency, orthographic neighborhood density, and morphological complexity or word length, also shape symptom expression. For instance, Italian speakers with surface dyslexia show stress assignment errors, reflecting Italian's prosodic patterns and impairments at multiple levels of the reading process.^14^ By systematically considering morphosyntactic, lexical‐semantic, and phonological dimensions across spoken and written modalities and across languages, such an integrated cross‐linguistic linguistic profiling approach promotes the development of culturally and linguistically appropriate tools to more accurately characterize language impairments in multilingual individuals with neurological communication disorders.

To capture the complexity of multilingual language use, a critical component of assessment is the comprehensive documentation of language history and proficiency across all languages spoken by an individual. This information is essential for establishing a reliable baseline profile of an individual's language combinations and complements considerations of cross‐linguistic differences and linguistic typology. This dynamism is reflected in processes such as language attrition, the gradual loss of proficiency due to disuse,^15^ and cross‐linguistic influence, where features of one language affect another. Language history and proficiency data are typically gathered through self‐report questionnaires and, when appropriate, supplemented by caregiver or family member input to provide a longitudinal perspective on language acquisition and use. Such information is indispensable for interpreting performance patterns, disentangling individual differences in language learning trajectories, and evaluating the extent and nature of impairment. Ultimately, integrating language history with cross‐linguistic typological considerations not only enhances diagnostic precision but also informs more tailored, effective intervention planning for multilingual individuals, further discussed in Section 6.

In addition to typology and language history, diagnostic accuracy is further shaped by (a) assessment modality‐related factors and (b) suprasegmental linguistic features, including social and regional dialectal variations. Most existing assessment tools are designed for spoken languages, rendering them fundamentally inaccessible and inappropriate for the millions who use signed languages.^16^ Because signed and spoken languages differ markedly in their phonological (or cherological), morphological, and syntactic structures, they require the development of entirely distinct assessment paradigms rather than simple adaptations of spoken language tools. Even within spoken and written languages, the modality of stimuli is a critical factor. An individual's proficiency may vary considerably between auditory‐oral skills (listening and speaking) and visual‐written skills (reading and writing). Assessments that fail to differentiate between these channels risk misinterpreting a modality‐specific difficulty as a generalized language impairment. Additionally, the influence of social and regional dialects and code‐switching, or the systematic mixing of languages, further complicate assessment. Standardized assessments often penalize these natural variations, which can significantly affect performance outcomes.^17^ A comprehensive assessment must therefore reflect the full spectrum of an individual's linguistic experience prior to symptom onset to avoid misattributing dialectal variation or language mixing to disordered language functioning. Capturing this complex linguistic reality is no small task. However, navigating this linguistic landscape requires a nuanced understanding of the diverse ways in which language is structured and used and how it evolves across different sociolinguistic contexts.

## 3 COGNITIVE DIFFERENCES BETWEEN MONOLINGUALS AND MULTILINGUALS: IMPLICATIONS FOR ASSESSMENT

Without a clear understanding of the unique cognitive architecture of the multilingual mind, performance may be misinterpreted, resilience overlooked, and deficits misattributed. Despite its widespread prevalence, multilingualism remains insufficiently integrated into many research paradigms and clinical frameworks. Over the past two decades, research has increasingly focused on the so‑called *bilingual advantage*, with studies exploring its impact on a variety of cognitive domains. However, more recent work has moved toward a more nuanced and context‐dependent view, emphasizing variability across individuals, languages, tasks, and sociolinguistic environments.

This shift in perspective is critical, as managing multiple languages is thought to shape both functional and structural brain networks over time, with consequences for cognitive efficiency and resilience across the lifespan. The neural underpinnings and shared networks supporting these adaptations are discussed further in Section 4. Early research suggested that multilingualism had also been associated with improved executive functions,^18^, ^19^ attentional control,^20^ increased sensitivity to novelty,^21^ and exploratory behavior.

Beyond attentional and executive functions, navigating multiple linguistic codes often demands heightened metalinguistic awareness^22^ and sensitivity to social and pragmatic cues,^23^ with possible transfer to non‐linguistic domains such as moral reasoning and theory of mind.^24^ However, the robustness of these findings has been called into question. Recent meta‐analyses and replication studies have challenged these findings, often reporting null effects when controlling for publication bias and confounding factors.^25^, ^26^ In addition, some studies report a so‐called *bilingual cost*, whereby multilingual speakers show reduced performance on certain language measures (e.g., lexical retrieval or verbal fluency) compared to monolinguals, particularly in the dominant language.^27^ The inconsistencies may reflect not only methodological variability but also true differences in how multilingualism affects different subgroups of multilinguals, shaped by individual factors such as socioeconomic status (SES), cultural background, and immigration history.^28^

Beyond the debated executive and attentional benefits, multilingualism exerts a consistent influence on higher‐order cognitive functions, particularly social cognition. A meta‐analysis by Xia and Haas^29^ revealed that both bilingualism and multicultural experience were positively associated with enhanced social‐cognitive abilities, notably in intergroup perception and perspective‐taking. This suggests that exposure to multiple languages and cultural contexts fosters a more nuanced understanding of others and supports healthier and more empathetic intergroup relationships. Evidence also shows that bilinguals outperform monolinguals on tasks such as the *Faux Pas* test, indicating distinct patterns of social‐cognitive processing.^30^ This finding aligns with research in children showing that multilingualism influences social cognition, particularly by enhancing perspective‐taking, as exposure to multiple languages fosters more effective understanding of others’ intentions.^31^ Given that certain neurological conditions, such as the behavioral variant of frontotemporal dementia and traumatic brain injury, can present with early impairments in social cognition, understanding how multilingualism shapes these abilities is therefore crucial for accurate assessment and intervention planning. Moreover, the observed association between multilingualism and divergent thinking suggests a potential expansion of the cognitive advantage hypothesis, extending its relevance beyond executive and attentional functions to encompass flexible, creative, and socially attuned forms of thinking. However, these findings warrant further replication and confirmation, ideally through large‐scale studies, before firm conclusions can be drawn.

Finally, multilingualism also offers protective effects in aging, particularly through the mechanism of cognitive reserve. Defined as the brain's capacity to maintain function despite pathological changes, cognitive reserve has been shown to be greater in multilinguals. That is, dementia symptoms typically emerge 4.1 years later in bilinguals than in matched monolinguals with comparable levels of neuropathology,^32^ and these results have even been observed in indigenous populations.^33^ This delay is attributed to enhanced executive and attentional control, which support compensatory cognitive strategies. Memory performance also appears to benefit from multilingualism: Bilingual older adults perform better in episodic recall tasks, especially when the second language was acquired early and used consistently throughout life.^34^ However, the overall evidence remains mixed. A recent meta‐analysis did not find consistent support for a protective effect of multilingualism on cognitive decline or dementia incidence.^35^ Notably, earlier positive findings often stem from retrospective studies, potentially confounded by education or cultural differences in dementia care access, complicating causal interpretation. As such, while multilingualism may contribute to cognitive resilience, especially in specific domains or populations, more longitudinal and methodologically rigorous studies are needed to clarify its role in aging and dementia.

## 4 NEURAL NETWORKS OF LANGUAGE(S): DECODING MULTILINGUAL BRAIN DYNAMICS

Neglecting the distinct neural dynamics of the multilingual brain risks misreading resilience as pathology and overlooking therapeutic intervention. Advances in neuroimaging and electrophysiological methods reveal that multilingualism affects both the structure and function of the brain in complex ways.

Structural neuroimaging, for instance, has shown that multilingualism is associated with increased gray matter density in regions such as the inferior parietal cortex, a change linked to the demands of learning and managing a new vocabulary.^36^ Furthermore, diffusion tensor imaging (DTI) studies reveal enhanced microstructural integrity in white matter tracts, including the corpus callosum, suggesting strengthened inter‐hemispheric communication pathways.^37^

On a functional level, functional magnetic resonance imaging (fMRI) and positron emission tomography (PET) studies demonstrate that while multiple languages recruit largely overlapping neural networks, the specific pattern of activation is modulated by factors such as proficiency and age of acquisition (AoA). A key finding from a meta‐analysis of functional neuroimaging studies suggests that while first language (L1) lexico‐semantic processing relies on a widespread cortico‐subcortical system, second language (L2) processing often recruits additional regions associated with executive control, particularly when L2 is acquired later in life or is less proficient.^38^ This increased demand on control networks is also evident in the brain's intrinsic architecture. Resting‐state fMRI studies show that multilinguals exhibit enhanced functional connectivity compared to monolinguals, with recent evidence pointing to particularly strong integration between the cerebellum and left frontal cortex in early language learners.^39^ This suggests that the experience of managing multiple languages optimizes communication pathways across the brain, fostering more efficient neural organization.

Bilingual language control (BLC) involves maintaining language separation and selecting the context‐appropriate language.^40^ A meta‐analysis of voluntary switching tasks identified consistent activation in eight regions, including the left inferior frontal gyrus, middle temporal and middle frontal gyri, pre‐supplementary motor area, bilateral caudate nuclei, and right precentral and superior temporal gyri.^41^ These areas overlap with domain‐general executive networks, implying that BLC is an extension of broader control systems.

While fMRI provides excellent spatial resolution, electroencephalogram and event‐related potential (ERP) studies offer the temporal precision needed to track multilingual language processing in milliseconds. This research has identified a cascade of neural responses sensitive to different aspects of language that are strongly modulated by a multilingual's linguistic experience. Factors such as AoA and proficiency are critical. For example, in the domain of syntax, early and highly proficient L2 learners often display a native‐like ERP pattern (e.g., a left anterior negativity followed by a P600) in response to grammatical violations, whereas late or less proficient learners may show delayed, reduced, or qualitatively different neural responses.^42^ Critically, this neural convergence is further constrained by linguistic typology. A study by Diaz and colleagues^43^ demonstrated that native‐like ERP patterns were more likely to emerge for L2 grammatical structures that are also present in the learner's L1. Syntactic features unique to the L2, however, continued to elicit non‐native‐like neural responses even in highly proficient early bilinguals. This highlights a crucial point: behavioral fluency does not always equate to native‐like neural processing. An L2 speaker may achieve perfect accuracy on a grammaticality judgment task while their brain continues to process the information in a manner distinct from a native speaker, a subtlety that only high‐temporal‐resolution neuroimaging can reveal.

The brain's capacity for language learning is not uniform across the lifespan but is shaped by sensitive periods of heightened neuroplasticity, during which neural circuits are maximally receptive to environmental input.^44^, ^45^ Multilingualism serves as a powerful natural experiment for studying this experience‐dependent plasticity. The process of acquiring and using multiple languages induces lasting structural adaptations, including increased gray matter density in key language‐related cortical areas and enhanced microstructural integrity and myelination of white matter tracts that support efficient neural communication.^37^ These adaptations reflect the brain's dynamic ability to reconfigure itself in response to sustained cognitive demand. Just as the brain adapts to language acquisition, it is theorized to reorganize in response to language disuse, a process known as attrition. The neural mechanisms of language attrition are less understood than those of acquisition, but they are thought to involve a dynamic interplay of network reorganization and cross‐linguistic influence rather than a simple “loss” of knowledge. While direct neuroimaging evidence is still emerging, it is hypothesized that prolonged disuse of a language may lead to functional changes, such as reduced activation in its core processing network, and potentially trigger compensatory reorganization, where other networks (e.g., in the right hemisphere or domain‐general control regions) are recruited during retrieval attempts. Electrophysiological studies could, in principle, track these changes with high temporal resolution, potentially revealing how the attrited L1 is processed through the lens of the now‐dominant L2 grammar.^46^ A significant methodological challenge in this area of research, however, is the difficulty of disentangling the neural effects of language attrition from the natural cognitive and neural changes associated with healthy aging, as these two processes are often confounded in immigrant populations who are the typical focus of attrition studies. This underscores that the multilingual brain is not a static entity but is continuously and dynamically shaped by linguistic experience across the lifespan.

In conclusion, multilingualism profoundly shapes the brain's structure, function, and adaptability. Overlapping but flexible networks, reliance on domain‐general control, electrophysiological sensitivity to AoA and proficiency, and the ongoing neural reorganization seen in both acquisition and attrition all underscore the unique dynamics of the multilingual brain. Understanding these processes is critical to avoid misdiagnosis and recognize compensatory mechanisms.

## 5 CLINICAL CROSSROADS: UNMASKING BIAS IN LANGUAGE ASSESSMENT

If multilingualism is not considered, clinicians risk overlooking linguistic, cultural, and psychometric variables, leading to bias, misdiagnosis, and inadequate care (Table 1). This risk is further amplified when languages are assessed in isolation, implicitly treating multilingual individuals as multiple separate monolinguals, rather than as speakers with a distributed and interacting linguistic repertoire. A holistic assessment approach instead recognizes cross‐linguistic interactions and the complementary distribution of skills across languages. Relying on monolingual‐centric tools remains a pervasive issue in current clinical practice. For instance, commonly used aphasia batteries like the Boston Diagnostic Aphasia Examination (BDAE‐3) and the Western Aphasia Battery are, in some languages, direct translations from English, without adjustments for language‐specific features and culture.^47^ This section addresses two major challenges: (1) the adaptation of tests to account for linguistic differences within and across a multilingual's full language repertoire and efforts to harmonize language assessments across languages and (2) the complexities of test administration, including scoring difficulties and procedural inconsistencies when working with multilingual individuals.

**TABLE 1:** Comparison of monolingual‐centric versus multilingual‐appropriate language assessment practices.

	Monolingual‐centric practice	Multilingual‐appropriate practice
**Language background assessment **		Assessment of language‐specific domains (e.g., proficiency, age of acquisition, context of use, language dominance and preference) and education (literacy, education language, years of schooling, neurodevelopmental difficulties)
**Stimulus selection**	Usually appropriate if culturally matched, minimal bias if same L1 and culture	Stimuli might induce cultural bias, poor validity, unfamiliar vocabulary
**Normative data collection**	Norms relevant for monolinguals based on level of education, sex, age, and more	Monolingual norms might generate false positives (underperformance) or false negatives (compensation across languages), leading to inappropriate comparisons
**Test administration design**	Simple logistics, examiner usually fluent in native language	Upstream preparation (test selection, translation), risk of examiner bias, misunderstandings from participant, discomfort in non‐dominant language, fatigue from switching between languages, need for interpreters
**Scoring criteria definition**	Tasks reflect daily use; errors are more easily interpreted and map to disorder profiles	Underperformance due to language proficiency, code‐switching might be considered incorrect, help of interpreter trained in domain might be needed to help score errors
**Clinical interpretation**	Based on correct responses and comparing to monolingual norms	Misclassification of language transfer or code‐switching as errors, over‐pathologizing or missing true deficits, need of clinical analysis of errors instead of referral to English diagnostic criteria and norms
**Diagnostic decision**	Based on correct responses and norms, assessment of functional impact of language impairment	Interpretation based on patterns of all languages tested, comparison between languages, careful about misdiagnosis due to proficiency, assessment of functional impact of language impairment
**Clinical recommendations**	Assessment of functional impact of language impairment, evidence‐based practice in rehabilitation	Assessment of functional impact of language impairment, choose target rehabilitation languages, treatment on multiple languages allowing cross‐linguistic transfer

Some language tests aim to fill this gap by adapting to language and culture, such as the Comprehensive Aphasia Test,^48^ the Bilingual Aphasia Test,^49^ and the Quick Aphasia Battery^50^ for aphasia syndromes, the Naming Assessment in Multicultural Europe,^51^ the Multilingual Picture Naming Test for mapping language during awake surgery,^52^ or the Multilingual Naming Test.^53^

One of the central challenges in cross‐linguistic language assessment is the lack of direct equivalence between languages. As Ivanova and Hallowell^47^ emphasize, even closely related languages do not exhibit one‐to‐one mappings in vocabulary or syntactic structures. As a result, careful upstream preparation is required, including thoughtful test and stimuli selection,^54^ translation, adaptation, and administration planning, to minimize bias and patient misunderstandings. Without such preparation, assessment outcomes may be confounded by discomfort in a non‐dominant language, fatigue related to switching between languages, or reduced performance driven by limited proficiency rather than underlying impairment. Indeed, stimuli may induce cultural bias or include unfamiliar vocabulary, resulting in poor ecological and construct validity. Assessment based on monolingual norms may generate false positives (e.g., a Spanish–French bilingual assessed only in French may underperform on a naming or vocabulary task in their non‐dominant language, creating the false impression of a lexical deficit) or false negatives (e.g., a patient with early lexical retrieval difficulties in Spanish may appear unimpaired if they strategically switch to French during a verbal fluency task, masking the underlying impairment). Both scenarios can mislead clinicians and lead to inappropriate diagnosis. This complicates comparisons of language performance across a multilingual's languages, requiring both solid theoretical grounding and methodological adaptation.

For cross‐linguistic assessment, it is important to control (psycho)linguistic variables such as word frequency, imageability, AoA, word length, orthographic regularity, sentence complexity, and cultural context.^55^ However, this is often challenging in under‐represented languages due to limited corpora and high variability in linguistic features. Additionally, differences in phonology, morphology, syntax, orthography, and semantics can significantly alter task demands, making raw score comparisons misleading.

Assessment practices must therefore account for typological incongruities and language dominance. These issues raise fundamental questions about how impairment is defined and measured across languages, even within the same individual, and underscore the need for frameworks that reflect the multidimensional nature of multilingual language use. To address these complexities, clinicians and researchers should clearly identify the cognitive and/or linguistic domain under investigation and integrate (psycho)linguistic variables. Education‐related factors, including literacy, schooling language(s), years of education, and history of neurodevelopmental difficulties, are equally critical to consider. Finally, multilingualism history should be considered an essential component of multilingualism assessment. While proficiency, AoA, and dominance capture the current state of multilingual abilities, a history of language experience traces multilinguals’ development and change over time, providing essential context for interpreting assessment results.

The recent neuropsychological adaptation of the International Test Commission Guidelines for Translating and Adapting Tests (Second Edition)^56^ highlights critical considerations for translating and adapting cognitive tests across languages and cultures. These include assessing the construct validity of the assessment in the target population and emphasizing functional rather than literal equivalence. Translation should involve multilingual experts and be supported by empirical validation, including pilot testing and statistical analyses to ensure reliability and construct equivalence. In this context, it is important to note that back‐translation, although widely used, is a suboptimal procedure to establish test‐translation adequacy^57^ because it does not guarantee functional, cultural, or conceptual equivalence needed for valid language and neuropsychological assessments. Finally, standardized administration procedures and adapted interpretive frameworks are essential to avoid introducing bias. These steps are particularly vital in the context of multilingual assessment, where linguistic and cultural differences can significantly distort test performance and threaten the validity of diagnostic conclusions. Ultimately, interpretation should include analysis of functional language impairments across languages, which can guide clinical decisions such as the selection of treatment languages and the potential for cross‐linguistic generalization in rehabilitation.

Another major challenge is that most patients are assessed only once, in a single language, and their performance is compared to existing monolingual norms. Clinicians are often left wondering what to do with answers provided in another language, answers that clearly reflect knowledge and ability but that are not accounted for in standard scoring. In these cases, clinical analysis of errors is essential to distinguish between true deficits and features of multilingual language use. This leads to significant interpretive difficulties and further highlights the need for multilingual norms and comparative assessment strategies that account for a patient's entire linguistic repertoire. Such strategies should aim to compare performance across languages, identify potential transfer effects, and assess the functional impact of language impairments in real‐world settings, ultimately guiding treatment in one or multiple languages based on patient needs and linguistic context.

In addition to formal language assessment tools, questionnaires also provide valuable self‐reported information on language background and use.^58^ The Language Experience and Proficiency Questionnaire (LEAP‐Q),^59^ Language History Questionnaire (LHQ 3.0),^60^, ^61^ Language and Social Background Questionnaire (LSBQ),^62^ Bilingual Switching Questionnaire,^63^ Ecological Momentary Assessment (EMA),^64^ and Bilingual Language Profile (BLP)^65^ constitute non‐exhaustive examples of questionnaires or methods used in clinical practice. However, these questionnaires capture only certain aspects of multilingualism, often emphasizing proficiency and AoA and overlooking context of language learning (formal instruction vs immersion), patterns of use over time, and/or language switching. Some questionnaires try to gather such information, specifically in relation to language use and exposure within the sociolinguistic environment, such as the Contextual Linguistic Profile Questionnaire (CLiP‐Q).^66^ It is important to note that many of these tools, including the widely used LEAP‐Q, were not originally developed for clinical populations and may not be easily accessible for individuals with neurological conditions. In contrast, other questionnaires like the BLP and LHQ are generally easier to administer and complete, which may improve the accuracy and reliability of responses in clinical contexts. Therefore, caution is needed when determining what questionnaire data are considered essential versus supplementary. While incorporating such tools is valuable, they should always be supplemented with clinical interviews and contextualized assessments that are adapted for cognitive and linguistic accessibility, to better account for factors such as fatigue, discomfort, or misunderstandings, particularly when assessments are conducted in a non‐dominant language.

In test administration, the absence of interpreters or translators places speakers of non‐dominant languages at risk of exclusion from public discourse and denial of essential services. Yet, relying on professional or family interpreters does not fully resolve the problem. Translation can result in information loss, and family members may intervene by intentionally or unintentionally guiding, assisting, or rephrasing responses when the patient struggles. They are also less likely to use precise clinical terminology, and patients often report lower satisfaction with such arrangements.^67^ Moreover, critical clinical and linguistic markers, such as syntactic impairments, semantic deficits, or paraphasias, may go unnoticed by interpreters unfamiliar with their diagnostic significance. Interpreter‐mediated sessions further complicate error scoring: Code‐switching or circumlocutions may be judged as incorrect, while genuine deficits can be overlooked or underestimated. These issues risk oversimplification or mistranslation, undermining the validity of the assessment. Finally, involving family members introduces an additional concern: the potential compromise of patient confidentiality.

The European Consortium on Cross‐Cultural Neuropsychology recommends systematic use of trained interpreters, pre‐assessment planning, culturally appropriate tools, and careful management of communication dynamics.^6^ However, in highly multilingual societies, such as India or sub‐Saharan Africa, the primary challenge lies in designing assessments that are intrinsically adapted to local contexts rather than relying on interpreter‐mediated testing.^68^

During assessment, clinicians should manage communication dynamics to minimize misinterpretation and ensure clarity and understanding. Interpretation of results must account for the potential impact of interpreter mediation on test performance. Finally, there is a need for clinician training and further research to standardize practices in this complex but increasingly common clinical context. Locally developed guidelines and culturally embedded practices are therefore crucial, and global frameworks must remain sensitive to these distinctions when proposing universal recommendations.

While methodological inconsistencies and limited linguistic representation remain pressing issues, they are likely symptoms of a deeper challenge: the need to reconceptualize our assessment paradigms altogether. Traditional tools often fail to capture the dynamic and adaptive nature of the multilingual mind. Although some clinical guidelines and practice recommendations exist (e.g., from professional bodies such as the American Speech‐Language‐Hearing Association), they are often developed for narrowly defined populations or specific linguistic contexts, limiting their applicability and generalizability to the heterogeneous experiences of multilingual adults. This calls not only for more inclusive and culturally adapted versions of existing tests, but for fundamentally different types of assessments that are sensitive to the unique experiences, processing strategies, and language control mechanisms of multilingual individuals. Future efforts must consider how phenomena such as language switching, cross‐linguistic transfer, and context‐dependent proficiency shape communicative performance. Rather than treating multilingualism as a variable to control for, we should treat it as a lens through which cognitive and linguistic functioning can be better understood. By embracing more ecologically valid paradigms and designing tools grounded in the lived reality of multilinguals, we aim to advance both clinical accuracy and theoretical insight.

## 6 ASSESSING THE UNASSESSED: LINGUISTIC, EDUCATIONAL, AND SOCIAL BIASES IN LANGUAGE EVALUATION

Failing to address linguistic diversity and intersectional realities renders vast populations' voices unheard, exacerbating health inequities and undermining diagnostic validity. As discussed in previous sections, most language assessment tools are developed in English or closely related languages, creating a significant gap for speakers of under‐represented languages.^9^ In the context of increasing global migration, this raises essential concerns about inclusivity and diagnostic equity. The overrepresentation of WEIRD (Western, Educated, Industrialized, Rich, Democratic) populations in behavioral research^69^ limits generalizability and perpetuates clinical disparities. Greenfield argues that cognitive and intelligence tests are shaped by the cultural context of their origin, reflecting the norms, knowledge, and values of Western societies.^70^ Consequently, applying these assessments in different cultural settings requires ensuring that each test item and its expected responses carry equivalent meaning and value across cultures. This means that the very foundation of psychological and neuroscientific knowledge is built on a narrow demographic, leading to tools and practices that are not universally applicable.

Beyond geographic and cultural bias, significant issues arise from educational and literacy disparities. Normative data for many commonly used tests are largely drawn from highly educated samples (88% from university‐based populations^71^), and in many Western countries, validation has often been limited to highly educated multilingual groups,^72^ restricting their applicability to broader populations. This is problematic given that over 750 million people worldwide are illiterate.^73^ Furthermore, test formats such as black‐and‐white line drawings (e.g., in the Boston Naming Test) may not be appropriate for individuals with limited education, suggesting a need for alternative formats such as real object naming.^72^ Although a systematic review has examined neuropsychological assessments for dementia in low‐educated, non‐Western, and illiterate populations,^74^ no comparable review exists for language assessments across all neurological conditions, despite clear evidence that literacy significantly influences language test performance.^75^ SES may also influence language outcomes. Matić Škorić and colleagues^76^ showed that while SES had limited impact on language performance in Norwegian participants, it significantly affected scores among Croatian participants with and without aphasia because of a lower SES. These findings demonstrate that education, literacy, and SES must be considered even when comparing populations from similar geographic regions.

Other dimensions of identity, such as disability, gender identity, and migration history, also warrant attention. Gender‐diverse individuals are often marginalized in clinical settings and research due to binary response options and exclusionary language. Adopting gender‐inclusive practices, including neutral language and expanded identity categories, improves both participation and assessment validity.^77^ Similarly, individuals with language disabilities may require adapted testing procedures to ensure accessibility and fairness. In the case of “forced” multilinguals, individuals who acquire additional languages through displacement or migration, language use is often shaped by necessity rather than choice. These complex and unbalanced profiles differ from elective multilingualism and must be interpreted in light of the individual's sociolinguistic and migration history.

Frameworks such as ECLECTIC (E: education and literacy; C: culture and acculturation; L: language; E: economics; C: communication; T: testing situation: comfort and motivation; I: intelligence conceptualization; and C: context of immigration)^78^ offer valuable guidance for clinicians and researchers striving to conduct ethically sound, valid, and inclusive neuropsychological assessments that reflect the full complexity of individuals' lived experiences. Indeed, a substantial body of research has highlighted that individuals from ethnic minority groups are more likely to receive false‐positive diagnoses in neuropsychological assessments.^79^ Testing situations may also be uncomfortable, unfair, and culturally inadequate for some ethnic minorities, as they often fail to reflect the individual's cultural norms, perspectives on intelligence, and values.

In sum, the assessment of multilinguals demands careful consideration of intersecting aspects of identity, including cultural background, education, literacy, language experience, and broader social positioning. Table 2 outlines how these factors shape multilingual language assessment. As highlighted by Kamaldeep Bhui and colleagues,^80^ individuals from marginalized groups are disproportionately exposed to social disadvantage yet remain systematically under‐represented in research, limiting their access to advances in clinical science. Relying on monolingual, Western‐based tools without appropriate adaptation can lead to misdiagnosis, reinforce systemic inequities, and further restrict access to appropriate care for those least likely to benefit from scientific progress. An inclusive approach to assessment and research therefore represents not only a methodological imperative but an ethical responsibility, grounded in principles of justice and fairness, ensuring that advances in science benefit all populations rather than a privileged subset. Failure to anticipate barriers related to literacy levels and linguistic isolation risks excluding entire groups unless measures and procedures are adapted, optimized, and tested in advance, with adequate resources allocated for translation and interpretation. This further requires building sustained community partnerships and networks of trusted organizations, as well as adapting research infrastructures and study designs to be culturally responsive and to address epistemic injustices. Ultimately, assessment is not an end in itself but the foundation for diagnosis and intervention. When it falls short, treatment decisions risk being misguided, with lasting consequences for patients.

**TABLE 2:** Impact of linguistic, sociodemographic, and identity factors on multilingual languages assessment.

Category	Factor	Impact and implications for assessment
**Linguistic**	***Language modality***	Assessments designed for spoken languages are inaccessible for signed languages, necessitating fundamentally different testing paradigms, not just simple adaptations.
	***Stimulus modality***	Proficiency can differ between oral/auditory and written/visual skills. Modalities must be assessed separately to avoid misinterpreting a modality‐specific issue as a general impairment.
	***Typological distance***	Structural differences between languages (e.g., syntax, morphology) affect difficulty and cross‐linguistic transfer. Direct translation of tests is inadequate.
	***Dynamic proficiency***	Language skills are not static due to attrition and cross‐linguistic influence. Assessment should be longitudinal to distinguish natural changes from pathological decline.
	***Sociolinguistic variation***	Standardized tests often penalize common practices like code‐switching and dialectal variations as "errors." Assessments must be sensitive to the individual's specific linguistic context.
**Sociodemographic**	***Education and literacy***	Normative data are often biased toward highly educated populations, and test formats may be inappropriate for individuals with low literacy. This requires using alternative formats and considering literacy levels in each language.
	***Socioeconomic status (SES)***	SES can influence language performance. It must be considered during interpretation and when selecting comparison groups.
	***Cultural background***	The assumption of cultural universality in tests can lead to misinterpretation of communication patterns. Tools must be culturally sensitive and designed with multicultural input.
	***Migration history***	"Forced" multilingualism (due to migration) creates different language profiles than elective bilingualism. Assessments must be interpreted in light of the individual's sociolinguistic history.
	***Disability***	Standard procedures may be inaccessible. Adapted materials and processes are required to ensure fair and equitable assessment.
**Identity**	***Gender identity***	Binary response options and exclusionary language can marginalize participants. Assessments should use gender‐neutral language and inclusive categories to improve validity and participation.

## 7 RECLAIMING COMMUNICATION: TAILORED REHABILITATION FOR MULTILINGUAL APHASIA

Speech and language intervention in multilingual individuals with aphasia, whether post‐stroke or neurodegenerative (PPA), requires nuanced consideration of language use, communicative needs, and multilingual language representation. Theoretical and neural frameworks suggest that shared linguistic networks across languages enable cross‐linguistic transfer of therapy effects, particularly when languages are typologically similar or share cognates.^81^

Recovery patterns are influenced by lesion characteristics, AoA, proficiency, frequency of use, and sociocultural context. For instance, Ribot's law suggests better recovery in L1, while Pitre's law emphasizes premorbid proficiency. Empirical support comes from Kuzmina and colleagues,^82^ who highlight that in individuals with aphasia, the L1 is generally better preserved. However, when both languages are acquired before the age of seven, performance tends to be more balanced between L1 and L2, with language proficiency having only a modest influence. Other factors such as SES and cognitive reserve may also shape initial aphasia severity or recovery trajectories.^83^

Despite this, evidence‐based guidelines for multilingual aphasia rehabilitation remain scarce, especially regarding therapy duration and intensity. The current literature offers no consensus on optimal treatment intensity or language target for multilinguals, as established for monolinguals.^84^ Therefore, language choice for therapy often depends on clinician proficiency and material availability rather than on individualized linguistic profiles.

Empirical evidence from post‐stroke aphasia shows that therapy in one language can generalize to another, particularly when treatments target semantic features or use cognates or when the non‐dominant language is treated.^85^ However, outcomes vary depending on factors such as language similarity, type of impairment and therapy (semantic vs phonological), and a particular individual's language history.^86^

In PPA, the evidence base remains limited to a few case studies and small group studies. Montagut and colleagues^87^ demonstrated gains in lexical retrieval and script production across both treated and untreated languages in bilingual individuals with PPA, particularly when interventions leverage residual linguistic knowledge and strategic self‐cueing. Improvements were sustained at follow‐up, with some generalization to untrained items or scripts. Similarly, Lerman and colleagues^88^ observed cross‐linguistic gains from single‐language interventions in participants with PPA, though outcomes varied by language pair and individual profile. Barriers such as lack of multilingual clinicians or culturally adapted materials necessitate collaborative care models involving interpreters, multilingual staff, and teletherapy options.

In sum, while evidence for multilingual intervention is limited, available studies support multilingual approaches targeting functionally relevant languages. Cross‐language generalization is possible but not guaranteed and is shaped by numerous linguistic and individual factors. Future research should explore treatment mechanisms across diverse language pairs and contexts, optimizing care for an increasingly multilingual population.

## 8 ARTIFICIAL INTELLIGENCE (AI) AS ALLY? THE PROMISE AND PERILS OF MACHINE LEARNING AND GENERATIVE AI IN MULTILINGUAL TESTING

The integration of AI and machine learning (ML) into language assessment represents a fundamental shift from traditional psychometric approaches toward data‐driven, adaptive evaluation. ML models have already demonstrated the ability to outperform conventional scoring in multiple clinical domains, showing superior diagnostic accuracy for conditions such as aphasia and detecting early neurodegenerative changes through digital biomarkers imperceptible to human clinicians.^89^ The emergence of large language models (LLMs), driven by the sophisticated Transformer architecture,^90^ marks a further leap, offering unprecedented multilingual potential.^91^

The most transformative promise for multilingual populations may lie in the architectural design of these models. The language‐independent nature of embeddings in some LLMs means that information is stored based on semantic context rather than a specific language. This architecture could enable real‐time translation of instructions and accommodate natural code‐switching behaviors, a common practice poorly handled by monolingual paradigms, thereby providing a more accurate representation of an individual's true abilities.^92^ AI‐powered platforms could also offer flexible assessment delivery and objective scoring, reducing clinician bias and dependency on examiner availability.^93^ Indeed, promising tools such as the Toolkit to Examine Lifelike Language (TELL),^94^ Batchalign (https://github.com/TalkBank/batchalign2),^95^ and Redenlab (https://redenlab.com/) are being developed to provide scalable speech biomarkers, with some LLMs performing comparably to human evaluators in complex speech‐based tasks.^96^ Evidence from systematic reviews demonstrate that AI approaches to speech and language processing can effectively monitor Alzheimer's disease progression through automated analysis of linguistic features,^97^ while comprehensive surveys of AI techniques reveal substantial advances in Alzheimer's detection across diverse datasets and methodological approaches.^98^

However, the effectiveness of these models is not uniform, and their adoption without addressing inherent biases risks amplifying existing health disparities. A primary concern is algorithmic bias stemming from training data. LLMs exhibit significant performance gaps between high‐resource and low‐resource languages, often suffering from “dialectal blindness” where legitimate regional variations are misclassified as errors. This data scarcity problem is exacerbated for the very populations AI is claimed to help; the lack of large, digitized corpora for many indigenous, creole, and endangered languages often causes LLMs to “hallucinate” or invent meanings, posing significant clinical risks.

Crucially, this issue of cross‐linguistic variability may extend beyond language models to digital assessment more broadly. Emerging evidence suggests that language‐independent cognitive tasks can be affected by a user's linguistic background. A recent study found that while most digital cognitive metrics remain stable across different L1s, others demonstrate significant systematic differences.^99^ As digital platforms become central to dementia screening, this variability raises concerns about hidden bias, underscoring the need for rigorous validation across diverse language groups.

Finally, a significant research‐to‐clinical gap persists, complicated by practical and ethical hurdles. The most accurate AI systems often remain proprietary commercial products, while available open‐weight models may lack transparency regarding their training data. Furthermore, the real‐time generative nature of some AI systems can introduce inconsistency, compromising standardized validation.^100^ Addressing these limitations requires a comprehensive ethical framework that mandates diverse training data, cross‐validation with human expertise, and community‐based validation protocols. Only through such rigorous standards can AI‐based multilingual assessment achieve the precision and cultural sensitivity required for equitable healthcare. Table 3 summarizes key opportunities and challenges of emerging innovations.

**TABLE 3:** Opportunities and challenges of emerging innovations in multilingual language assessment.

Innovation	Aspect	Opportunities	Challenges
**AI and machine learning**	***Accessibility*** ***and reach***	On‐demand testing, scalability, examiner‐independence	Low‐resource gaps, proprietary tools, research‐to‐clinic gap
	***Bias mitigation***	Objective scoring, cultural pattern recognition	Data bias, stereotype reinforcement, hallucination risk
	***Accuracy*** ***and validity***	Superior diagnostics, speech biomarkers, human‐level performance (e.g., Redenlab, TELL)	Speed over accuracy trade‐offs, dialect sensitivity, consistency issues
	***Nuance*** ***and context***	Code‐switching support, semantic embeddings, context‐flexibility	Social/cultural variability, dialectal blindness
	***Implementation***	Personalization, flexible delivery, scalable deployment	Transparency gaps, confidence cascade, need for validation
**Open science practices**	***Data*** ***and tool sharing***	Reproducibility, FAIR principles, democratization, open‐access tools (PEBL, AutoPATT)	Data privacy, interoperability issues, quality control
	***Collaboration***	Interdisciplinary work, cross‐sector networks (e.g., INCLUDE, CAT, NWB), large‐scale projects	Coordination burden, academic silos, incentive misalignment
	***Innovation and development***	Open‐source ecosystems, adaptability, bottom‐up innovation (e.g., PEBL, Batchalign, TELL)	Sustainability, maintenance burden, long‐term viability of resources
	***Standardization***	Protocol consistency, cross‐linguistic comparability, equitable metrics	Resource‐intensive, rigid frameworks, risk of dominance bias
	***Transparency*** ***and rigor***	Preregistration, bias reduction, confirmatory versus exploratory clarity	Incentive misalignment, underuse of null/replication studies

## 9 OPEN SCIENCE AS SHARED LANGUAGE: FOSTERING COLLABORATION AND TRANSPARENCY

Progress in developing equitable multilingual assessment is hindered when research remains siloed and its outputs inaccessible. Embracing open science and collaborative practices is therefore not just beneficial, but essential (Table 3). Central to this approach is open access to research data, tools, code, and publications, which underpins reproducibility and facilitates cross‐linguistic research. To be truly useful, such data must adhere to the FAIR principles: Findable, Accessible, Interoperable, and Re‐usable.^101^ Without open sharing and standardization of data and tools, researchers and clinicians are forced to operate in isolation, unable to build upon each other's work, validate findings across diverse populations, or develop universally applicable assessments.

This call for openness stands in stark contrast to the current landscape of clinical assessment, where many foundational tools are commercialized. Key assessments, such as the BDAE‐3 and the Western Aphasia Battery‐Revised, are distributed by psychometric companies at a high cost. Access often requires expensive, specialized training, creating significant barriers for clinicians and researchers, particularly in under‐resourced settings. This is a critical paradox, as the initial development and norming of these tests are frequently conducted by academic researchers, yet the final products become proprietary and inaccessible. This commercial model perpetuates inequity and slows progress, directly opposing the goals of creating widely available and adaptable tools for multilingual populations.

This shift requires large‐scale, interdisciplinary collaboration. Among several emerging initiatives, networks such as Include (https://include‐network.com/), which focuses on multilingual aphasia, unite diverse expertise to build the shared infrastructure that no single lab could create alone. Other prime examples are ManyLanguages (https://many‐languages.com/), which facilitates large‐scale, multilingual studies of language, and the Neurodata Without Borders (NWB) initiative, which provides a standardized data format for neurophysiology data.^102^

Open science also provides a framework for navigating key methodological challenges. A central tension exists between standardization, which ensures comparability, and flexibility, which is required to accommodate diverse sociolinguistic histories.^103^ The creation and dissemination of open‐source tools, along with data sharing, allows the empirical validation of protocols across populations, helping to develop frameworks that are both methodologically robust and culturally adaptable. Freely available and adaptable resources democratize access to high‐quality assessment methods and encourage community‐driven contributions, fostering innovation.

Finally, open practices enhance research transparency and rigor. In multilingual research, small, heterogeneous samples can increase the risk of spurious findings. Preregistration, documenting hypotheses and analysis plans before data collection, is a powerful tool to mitigate this.^104^ By clearly distinguishing planned confirmatory analyses from exploratory findings, preregistration reduces bias and contributes to a more credible evidence base for the field.

## 10 TOWARD LINGUISTIC EQUITY: CHARTING A NEW COURSE FOR MULTILINGUAL ASSESSMENT

Achieving linguistic equity requires a paradigm shift, moving beyond monolingual norms to embrace a truly multidisciplinary, culturally sensitive, and patient‐centered approach. Given the complexity of multilingual language assessment, a multidisciplinary approach is essential. Collaboration among speech‐language pathologists, neurologists, (neuro)psychologists, and (socio‐ and psycho)linguists ensures a more comprehensive understanding of each patient's linguistic profile. Cultural sensitivity is equally crucial. Language is deeply intertwined with identity, culture, and lived experience; thus, assessments must be adapted to respect the patient's sociocultural background and avoid biased interpretations. A culturally sensitive, team‐based approach not only improves diagnostic accuracy but also enhances therapeutic relevance and patient engagement. The call for a “paradigm shift” implies that the current system is not just flawed but fundamentally misaligned with the reality of multilingualism. Incremental adjustments to existing monolingual tests or practices will not suffice; a complete re‐conceptualization of language assessment, built on collaboration and sensitivity, is needed.

Future research should prioritize the development of multilingual assessment tools that are normed across different linguistic and cultural groups and that are informed by clinicians. Longitudinal studies exploring language change across the lifespan in multilingual patients will be vital for understanding progression patterns in various neurological conditions. Additionally, integrating advanced neuroimaging techniques with language assessment could offer deeper insights into brain‐language relationships in multilinguals. A promising direction is the development of a standardized international protocol that integrates multilingual experiences, cognitive functioning, and neuroimaging data in patients with progressive neurological diseases. This open‐ended initiative seeks to build a growing cross‐laboratory dataset and clarify the role of multilingualism in cognitive reserve.^105^ Recently, some datasets have been created and made freely available, such as the multilingual picture dataset that gathers normative data for naming and familiarity of pictures in 32 languages.^106^ Clinically, there is a growing need for flexible intervention models that can adapt to a patient's shifting language dominance and functional communication needs over time.

While a rigid, one‐size‐fits‐all approach is incompatible with the realities of multilingualism, there is still significant value in developing therapy and management approaches that are as inclusive and accessible as possible. The development of such frameworks, however, is only the first step. To ensure efficacy, they must be rigorously tested across diverse linguistic and cultural groups. This balanced approach allows clinicians to draw from evidence‐based, inclusive strategies while upholding the core principle of patient‐centered care. The ultimate clinical decisions must be guided by each patient's unique language history, personal preferences, cultural identity, and life goals. By starting with validated, accessible frameworks, clinicians can more effectively co‐create meaningful and functional therapy goals with patients and their families. This will ensure that interventions are not only effective but also support patients’ quality of life, autonomy, dignity, and emotional well‐being.

## 11 THEORETICAL FRONTIERS: RESHAPING LANGUAGE MODELS FOR A MULTILINGUAL WORLD

Adhering to monolingual‐centric theoretical models constrains our understanding of language by failing to account for the dynamic, adaptive, and integrative nature of multilingual cognition. As demonstrated across the preceding sections, multilingualism is not merely an additive phenomenon but a qualitatively distinct condition that reshapes how language is represented, processed, and maintained in the brain. Rather than being compartmentalized into separate, static systems, multiple languages appear to be organized in flexible, overlapping networks shaped by experiential factors such as age and context of acquisition, frequency of use, and relative proficiency. These insights compel a shift from static, modular conceptions of language to models that conceptualize linguistic knowledge as fluid, distributed, and context‐sensitive. Such models must account for phenomena like code‐switching, cross‐linguistic transfer, and shifting language dominance, not as exceptions or complications, but as central features of language processing in multilingual minds. This perspective aligns with emerging views of language as a repertoire of dynamically activated resources, responsive to communicative demands and sociocultural contexts.

To advance the field, we propose the following avenues for theoretical innovation and interdisciplinary research, leading to emerging questions:

- **Develop dynamic network models of multilingualism**: Prioritize computational and neural network models that capture the non‐linear dynamics of multilingual language processing and test whether changes in network properties associated with exposure, attrition, or rehabilitation predict clinical outcomes.
- **Integrate ecological and systems‐based approaches**: Use frameworks from systems neuroscience and ecological psychology to model interactions between internal linguistic–cognitive systems and sociolinguistic environments, improving predictions of real‐world communication.
- **Establish multilingualism as a model for neuroplasticity**: Combine advanced neuroimaging with longitudinal data to examine how language pairs, learning contexts, and lifespan trajectories shape neural reorganization and cognitive reserve in aging and neurological disease.
- **Create clinically oriented multilingual models**: Move beyond adapted monolingual tools to develop diagnostic and rehabilitative models sensitive to cross‐linguistic transfer, and predict optimal therapeutic strategies based on language dominance, typological distance, and disorder profiles.

These clinical imperatives highlight the urgent need for coordinated international initiatives translating multilingual theory into practice. In this context, the Multilingual Aphasia Practices (MAP) project from the Collaboration of Aphasia Trialists (CATs) addresses key gaps in clinical care. Drawing on global survey data, speech and language therapists/pathologists (SLT/Ps) worldwide report a growing need for specialized training programs to manage multilingual aphasia, given that they receive little to no formal preparation to work with multilingual clients.^107^ Aphasia is consistently identified by practitioners as one of the most challenging disorders to assess and treat, particularly in multilingual neurogenic populations. This challenge is compounded by a lack of standardized, culturally appropriate assessment and intervention materials, with respondents reporting high levels of dissatisfaction regarding the availability of resources for minority and under‐represented languages.^108^ Together, these findings highlight the need for structured support and clear clinical guidance. In response, the MAP project aims to develop and disseminate evidence‐based best‐practice guidelines for multilingual aphasia assessment and treatment, informed by international expertise and cross‐linguistic research, to support equitable and clinically actionable care across healthcare systems.

In sum, multilingualism should be recognized not as a variable to control for but as a catalyst for theoretical advancement. By centering multilingualism in our models of language, cognition, and neurobiology, we can develop more comprehensive, inclusive, and ecologically valid theories, opening new pathways for understanding the human mind across linguistic and cultural boundaries.

## 12 LIMITATIONS AND FUTURE RESEARCH

As a narrative review, this work does not follow a systematic search strategy or include formal risk‐of‐bias assessment, and literature selection may therefore be influenced by publication, language, and disciplinary biases. Although targeted database searches and reference chaining were used, some relevant studies, particularly those in under‐represented languages or regional journals, may have been missed. These limitations were addressed by drawing on cross‐disciplinary sources, international guidelines, and recent systematic reviews and by assembling a multilingual, interdisciplinary author team to inform interpretation and recommendations.

Future research should prioritize systematic and meta‐analytic approaches encompassing a broader range of languages, clinical populations, and assessment paradigms, alongside empirical validation of multilingual frameworks to support actionable, evidence‐based clinical tools.

## 13 FINAL RECOMMENDATIONS

The following recommendations are crucial for both clinicians involved in the assessment, diagnosis, and treatment of multilingual individuals and researchers aiming to advance the field. The overarching principle is that multilinguals are not simply multiple monolinguals in one brain^109^; this understanding must guide every aspect of assessment and rehabilitation. While these recommendations represent best practices, practical constraints related to time, funding, and resource availability may limit their implementation. Nonetheless, clinicians and researchers should apply these guidelines wherever feasible, adapting them to local and individual contexts.

**For clinicians**:

- **Select language assessments carefully**: Choose culturally and linguistically appropriate assessment tools controlled for key (psycho)linguistic variables like word frequency, familiarity, and literacy demands, where available. When possible, assessment should rely on standardized tools that have been translated with the properties of the target language in mind, culturally adapted, and validated in the target language. For example, while passive sentences are commonly used to assess syntactic deficits in English, linguistically adapted assessments may prioritize alternative syntactic structures in languages such as Arabic, Hebrew or Malay, where different grammatical constructions are more informative for detecting syntactic impairment.
- **Use adapted and flexible materials**: Incorporate tools like colored pictures or real‐life objects, if adequate norms are available, from the individual's context to increase ecological validity and accessibility across different cultural, educational, and literacy backgrounds. Additionally, EMA methods have already been successfully implemented in low‐literacy and racially and ethnically diverse populations, including immigrant households and ethnic minority groups, to assess a range of everyday behaviors. Although these applications have not yet focused on multilingualism, EMA represents a promising avenue for assessing language use and multilingual practices in an ecological manner, with the added advantage of being adaptable and validated for use in minority populations.
- **Collect a comprehensive multilingual and sociocultural history**: Gather detailed information about a given individual's languages, including AoA, order, and context of acquisition, proficiency, daily use, exposure patterns, code‐switching, and emotional associations, using aphasia‐friendly language and materials. In parallel, document the individual's educational background, SES, countries of schooling, languages of formal instruction, and literacy levels across languages. The BLP questionnaire is well suited for self‐reported measures of multilingualism in clinical populations, as it is brief, easy to administer, and appropriate for individuals with cognitive or language impairments. However, self‐reported proficiency can vary across cultures, with some populations tending to rate themselves higher or lower than others. In addition, the BLP is limited to two languages and is currently available only in dominant languages, which may reduce its applicability in highly multilingual contexts; when self‐report is uncertain, objective measures such as oral proficiency interviews or picture‐naming tasks (e.g., MINT) should be preferred.
- **Document premorbid language use and disorder‐induced changes**: Investigate premorbid skills in each language across contexts (e.g., home, work), and for progressive conditions, changes in language use should be charted over time in consultation with family and friends. When interpreting test performance, clinicians must consider bilingual costs, such as slower lexical access and smaller vocabularies in each language, modulated by AoA, proficiency, and language dominance. If unaccounted for, these bilingual costs may increase the risk of misdiagnosis, particularly in late‐acquired or less‐dominant languages.
- **Apply clinical judgment during scoring and interpretation**: Determine how code‐switching is taken into account in scoring and interpretation, acknowledging that its role may vary across individuals and sociolinguistic contexts. Rather than assuming code‐switching to be either compensatory or pathological, clinicians should consider multiple sources of information, including self‐ and informant reports of pre‐morbid language use, as well as frequency and familiarity effects shaped by proficiency and societal norms. For example, when scoring word retrieval on a test normed only in the dominant language, responses should be scored and reported both with and without words produced in another language, allowing clinicians to distinguish strategic language use from true lexical access deficits.
- **Use appropriate comparison groups**: Whenever possible, avoid using monolingual norms. Instead, use available multilingual norms or interpret performance within the individual's specific multilingual context. However, if only monolingual norms are available, interpret results with caution.
- **Leverage telepractice and international networks**: Make available remote assessment tools and collaborate with international clinicians and researchers to access appropriate tests, normative data, and linguistic expertise. However, this recommendation may be less feasible for patients with lower levels of education due to limited access to computers.
- **Select functionally relevant languages for rehabilitation**: Prioritize the languages most useful in the patient's daily life, considering their preferences, identity, and communication needs. Therapy choices are also constrained by the languages spoken by the SLT/P. When the clinician does not speak the patient's preferred language(s), strategies such as involving trained interpreters, collaborating with family members, reaching out to a local or international network of SLT/Ps, or focusing on transferable skills across languages can help bridge this gap.
- **Monitor treatment generalization across languages**: Be aware that therapy in one language may generalize to another language, depending on factors such as structural overlap, proficiency, and cognitive load. Notably, if an L2 remains relevant for daily use, therapeutic gains in L2 may also transfer back to L1.
- **Advocate for multilingual‐inclusive tools, policies, and open‐science practices**: Support the development, validation, and dissemination of tools adapted for diverse linguistic populations, and promote inclusive and open practices within healthcare systems.
- **Training in multilingual clinical practice**: Develop and support specialized training programs focused on multilingual assessment and intervention. Currently, SLT/Ps receive little to no formal preparation to serve multilingual clients, with only a handful of programs offering specialization in multilingual populations. As a result, many clinicians rely primarily on interpreters or cultural brokers, ad hoc assessment modifications, or referrals to multilingual clinicians. While these strategies can be useful, they cannot replace core competencies in multilingual clinical decision‐making. Targeted training would improve diagnostic accuracy, promote equity in care, and better reflect the linguistic realities of clinical populations.

**For researchers**:

- **Standardize and deepen the measurement of multilingualism**: Routinely measure and report multifaceted aspects of multilingualism beyond AoA and proficiency, including context of acquisition, language use patterns, exposure, and sociolinguistic environment. The LEAP‐Q is the most widely used questionnaire in multilingualism research, facilitating comparability across studies, though more thoroughly validated instruments such as the LSBQ may offer stronger psychometric properties. Questionnaire choice should be guided by practical considerations, particularly language availability; in this respect, the LEAP‐Q has an advantage as it is available in approximately 20 languages. Regardless of the tool selected, it should be appropriately adapted and validated, and researchers should include a language background summary table in supplementary materials reporting mean dominance, exposure, and context‐of‐use scores to support future meta‐analyses.
- **Diversify research populations and create inclusive norms**: Actively recruit participants from non‐WEIRD backgrounds to improve generalizability. Develop and validate assessment norms and tools for populations with varied educational, literacy, and socioeconomic backgrounds. In addition, in countries where a sizable minority speaks a non‐dominant language, efforts should be made to create validated norms for language assessments, not only for the dominant language but also for non‐dominant languages. Grant proposals must explicitly budget for translation services and community liaisons to build trust and access within under‐represented linguistic communities, rather than relying solely on university subject pools.
- **Enhance methodological rigor**: Prioritize large‐scale, prospective, longitudinal studies. Systematically control for confounding variables like SES and immigration history, and design studies to disentangle language attrition from healthy aging. Where large‐scale samples are unfeasible, researchers should adopt robust single‐case experimental designs with multiple baselines to account for the high variability in multilingual aphasia recovery.
- **Inform theoretical language models**: Challenge traditional monolingual‐based models by developing more flexible, ecologically valid frameworks that account for cross‐linguistic interactions, adaptive representations, and sociolinguistic diversity.
- **Foster interdisciplinary, community‐based collaboration**: Engage linguists, clinicians, neuroscientists, (neuro)psychologists, psychometricians, medical doctors, and community stakeholders in collaborative research to develop standardized protocols and comprehensive insights.
- **Embrace FAIR data principles**: Promote the development of open‐source tools and platforms to democratize access for language resources. For example, by leveraging open repositories such as TalkBank.org, which provide shared, reusable corpora of naturalistic speech in multilinguals.
- **Implement pre‐registration**: Document research hypotheses, methodologies, and analysis plans before data collection to enhance transparency and reduce biases such as p‐hacking, HARKing, and publication bias. Platforms like OSF (https://osf.io/) and AsPredicted (https://aspredicted.org/) can help draft, finalize, and share preregistrations.
- **Address algorithmic bias in AI development**: When creating AI/ML assessment tools, use diverse training data with authentic multilingual speech samples and include multilingual perspectives on development teams to ensure cultural competency. Validation metrics must be stratified: Report sensitivity and specificity separately for each language subgroup (e.g., balanced bilinguals vs dominant bilinguals) rather than a single aggregate score, which often masks poor performance in minority groups.
- **Conduct rigorous clinical validation for AI**: Before clinical deployment, establish rigorous standards to demonstrate AI accuracy across diverse multilingual populations, including cross‐linguistic protocols and validation against human expertise.
- **Investigate optimal rehabilitation parameters**: Conduct systematic research on the ideal intensity, dosage, and duration of therapy for multilinguals with acquired and progressive aphasia to establish evidence‐based treatment plans.

## 14 CONCLUSION

Current clinical language assessment, overwhelmingly based on monolingual norms, is fundamentally inadequate for the world's multilingual majority. The assessment and treatment of multilingual individuals with acquired and progressive neurogenic disorders cannot be a mere adaptation of monolingual methods; it demands a paradigm shift toward new and culturally and linguistically sensitive tools and theoretical models. Embracing the complexity of the multilingual brain is an ethical imperative for equitable healthcare.

## CONFLICT OF INTEREST STATEMENT

The authors declare that they have no competing interests. Author disclosures are available in the Supporting Information.

## Supporting information




**Supporting Information**: dad270366‐sup‐0001‐SuppMat.pdf
